# ChemXTree: A Feature-Enhanced Graph Neural Network-Neural Decision Tree Framework for ADMET Prediction

**DOI:** 10.1021/acs.jcim.4c01186

**Published:** 2024-11-05

**Authors:** Yuzhi Xu, Xinxin Liu, Wei Xia, Jiankai Ge, Cheng-Wei Ju, Haiping Zhang, John Z.H. Zhang

**Affiliations:** † Shanghai Frontiers Science Center of Artificial Intelligence and Deep Learning and NYU-ECNU Center for Computational Chemistry, NYU Shanghai, Shanghai 200062, China; ‡ Department of Chemistry, New York University, New York, New York 10003, United States; § Department of Computer and Information Science, University of Pennsylvania, Philadelphia, Pennsylvania 19104, United States; ∥ Department of Materials Science and Engineering, University of Pennsylvania, Philadelphia, Pennsylvania 19104, United States; ⊥ Chemical and Biomolecular Engineering, University of Illinois at Urbana−Champaign, Urbana, Illinois 61801, United States; # Pritzker School of Molecular Engineering, The University of Chicago, Chicago, Illinois 60615, United States; @ Faculty of Synthetic Biology, Shenzhen Institute of Advanced Technology, Shenzhen 518055, China; ▽ Shanghai Engineering Research Center of Molecular Therapeutics and New Drug Development, School of Chemistry and Molecular Engineering, East China Normal University, 200062 Shanghai, China

## Abstract

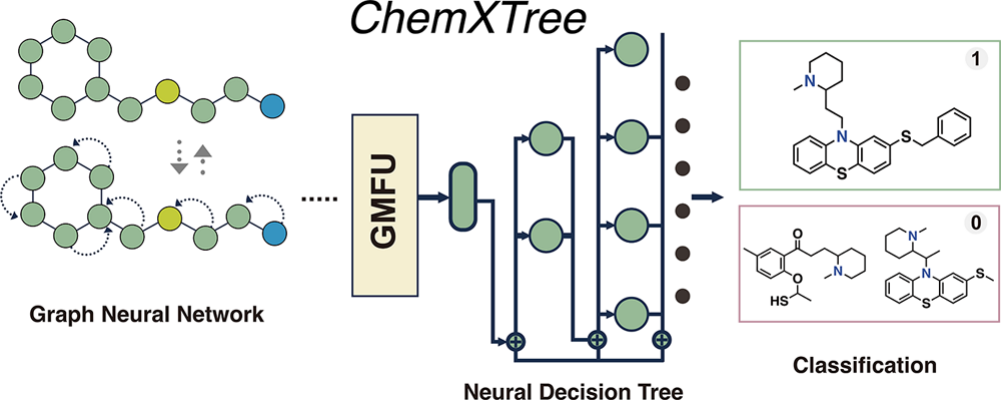

The rapid progression of machine learning, especially deep learning (DL), has catalyzed a new era in drug discovery, introducing innovative approaches for predicting molecular properties. Despite the many methods available for feature representation, efficiently utilizing rich, high-dimensional information remains a significant challenge. Our work introduces ChemXTree, a novel graph-based model that integrates a Gate Modulation Feature Unit (GMFU) and neural decision tree (NDT) in the output layer to address this challenge. Extensive evaluations on benchmark data sets, including MoleculeNet and eight additional drug databases, have demonstrated ChemXTree’s superior performance, surpassing or matching the current state-of-the-art models. Visualization techniques clearly demonstrate that ChemXTree significantly improves the separation between substrates and nonsubstrates in the latent space. In summary, ChemXTree demonstrates a promising approach for integrating advanced feature extraction with neural decision trees, offering significant improvements in predictive accuracy for drug discovery tasks and opening new avenues for optimizing molecular properties.

## Introduction

In recent decades, the development of machine learning has remarkably accelerated the pace of drug discovery.^1,2^ This acceleration is primarily because machine learning, unlike traditional drug discovery methods, can quickly process large data sets to identify crucial ADMET (absorption, distribution, metabolism, excretion, and toxicity) properties of drug compounds.^3−5^ By efficiently predicting factors like distribution coefficients, free energy, solubility, stability, etc., machine learning enhances the efficacy and safety assessment of potential drugs.^6,7^ As one of the most rapidly evolving subsets of machine learning, deep learning (DL) has had a huge impact on molecular property prediction. In molecular property prediction, DL methods avoids the need for predefined features, a limitation in traditional machine learning approaches.^8−10^ DL provides a direct mapping from input to output and categorizes its key methodologies for extracting molecular features into different types based on computational frameworks. Models that use visual grid encoding typically employ Convolutional Neural Networks (CNNs) to identify spatial features in molecules.^11−14^ Sequence-based models utilize architectures like Recurrent Neural Networks (RNNs) and Transformers to interpret molecular notations.^15,16^ Lastly, graph-based models apply Graph Neural Networks (GNNs) to understand the complex relationships between atoms and bonds in molecular structures.^17,18^

Recent advancements in deep learning (DL) for molecular property prediction have primarily focused on enhancing molecular representation capabilities.^11,19−22^ While these approaches have shown superior feature extraction compared to traditional molecular fingerprints,^23,24^ they have not consistently outperformed decision tree methods with simple molecular fingerprints on small data sets.^25−28^ Consequently, researchers have explored combining tree-based models with DL-based extractors to improve performance in various fields, including molecular prediction.^29,30^ Notable examples include XGraphBoost,^31,32^ which enhanced D-MPNN’s performance by replacing its FFN component with XGBoost, and similar approaches using Random Forest or XGBoost as output layers.^33−35^ However, these combination models often lack end-to-end training capabilities due to the absence of gradient back-propagation in traditional decision trees.^36^ To address this limitation, recent innovations have introduced Neural Decision Trees (NDTs).^36−38^ NDTs use gradient descent to optimize split points in continuous feature spaces, potentially enhancing the model’s ability to leverage complex information extracted by advanced DL methods.^39^ While promising, developing molecular property models using NDTs poses greater challenges than simple integration with traditional decision trees, making it a relatively unexplored area in the field.

Here, we propose ChemXTree, a feature-enhanced GNN-NDT Framework for molecular property prediction. To boost the feature selection capability, we introduce a new module named the Gate Modulation Feature Unit (GMFU) to refine and select the most informative features, serving as a bridge between feature extraction module and NDT. Following this enhancement, a differentiable NDT is integrated into the model as the predictive output layer. Extensive evaluations were conducted on benchmark data sets from MoleculeNet and eight supplementary drug databases.^40^ Results demonstrated that ChemXTree exhibits significant competitiveness with baseline models. Additionally, we conduct permutation experiments on the output layer and perform ablation studies on the GMFU, including the development of an LSTM-based variant, to assess their impacts on the overall model performance. Besides, in comparison to other representation methods, the latent space of ChemXTree’s GMFU output has already acquired the crucial pharmacological information necessary for substrate identification. In general, we demonstrated that even without pretraining, the combination model, ChemXTree, could compete with the performance of existing state-of-the-art(SOTA) models in classification tasks in small data sets. This approach is promising for broader application in smaller data sets with limited drug-related data availability.

## Results and Discussion

### ChemXTree Workflow

ChemXTree is specifically designed to address classification problems in small drug molecules. To achieve this, ChemXTree’s architecture utilizes Graph Neural Networks (GNN) for encoding molecules. The encoded output is then processed through a specially designed module, known as GMFU, which focuses on optimized feature selection. Following this, ChemXTree uses a Neural Decision Tree to further enhance the model’s classification capabilities.

**Figure 1:**
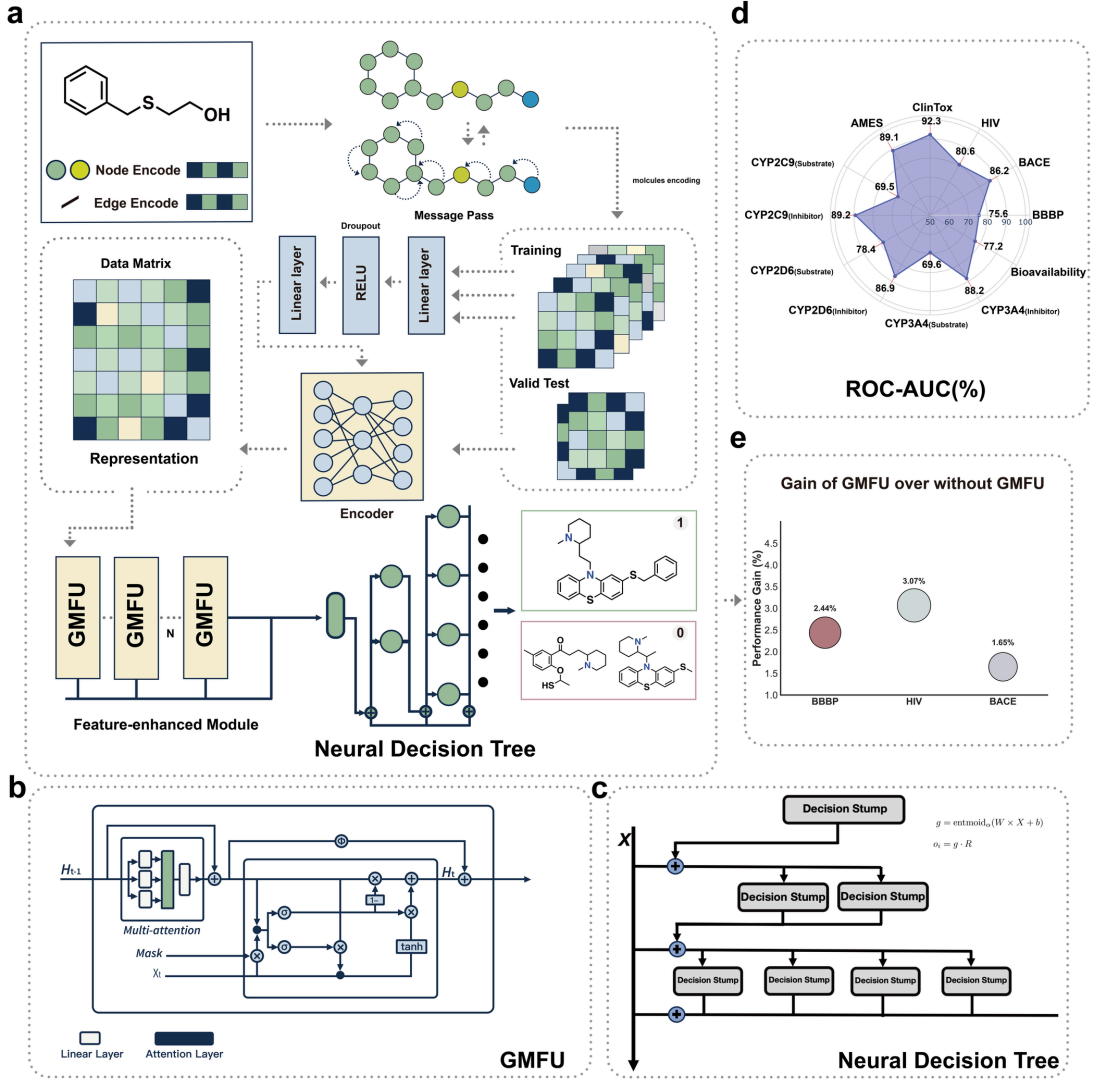
Workflow of ChemXTree: a combination model specifically engineered for the classification of small drug molecules. (a) The framework ChemXTree in training/testing process, including encoding module, GMFU modules, and the neural decision tree. (b) The structure of the GMFU module, illustrating the linear layers, attention layers, and the application of masks and activation functions. (c) The neural decision tree structure is (c), this is a soft decision process. (d) Performance of the ChemXTree across 12 benchmark data sets, showing ROC-AUC scores for different data sets like BBBP, BACE, HIV, and others in ChemXTree. (e) ChemXTree latent space shows a stronger clustering effect in distinguishing between active and inactive molecules in CYP2D6_Substrate testset, compared to other models.

To be more specific, as depicted in Figure 1a, ChemXTree initiates its process by first transforming the simplified Molecular Input Line Entry System (SMILES) of molecules into graphs. In these graphs, atoms, along with multiscale features such as charge and valency, serve as vertices (nodes), while chemical bonds act as edges. Subsequently, in the molecular representation learning stage, these initially encoded molecules are passed to Message Passing Neural Network (MPNN) for further processing and refining of the molecular encoding.^41^ Upon completion of the encoding training, the MPNN equips with trained weights and then functions as an encoder, encoding the validation and test sets, respectively. With this method, the encoded representations of the molecules in training, validation, and testing sets are obtained. To enhance the robustness of the molecular classification, we employ an ensemble approach. Specifically, the final molecular encoding is obtained by summing the outputs of five independently trained models for each molecule.

With the encoded molecular representations, ChemXTree aims to intensify the identification of task-relevant features for downstream tasks. To achieve this, we employ a network architecture composed of a series of GMFUs. Inspired by Gated Recurrent Units (GRU) and other variants like Gated Adaptive Network using similar gated mechanism, GMFU is designed for further feature selection.^42−44^ Specifically, each GMFU starts with self-attention on the input features, which are then fed into a gated structure equipped with reset and update gates to generate candidate feature representations (Figure 1b). The final output of the GMFU is computed by combining the previous hidden states with the current candidate features based on the update gate. Multiple GMFUs can be cascaded in a hierarchical fashion, where the output of one unit serves as the input for the subsequent unit. Different layers of GMFU engage in distinct feature selections, enabling the model to capture molecular representations at various levels of abstraction. This feature facilitates the integration of both global and local information into the final feature representation.

These features are subsequently fed into a differentiable neural decision tree for further employment (Figure 1c). To enable the latter optimization and backpropagation of gradients, our decision tree employs soft decisions that output continuous probabilities, as opposed to traditional hard and deterministic decisions that only yield 0 or 1. We leverage an ensemble of different differentiable trees whose outputs are weighted. This configuration leads to a stable and precise binary prediction result and ensures our model’s adaptability and effectiveness in handling high-dimensional molecular data spaces.

For more information about the architecture of ChemXTree, please see the Materials and Methods section.

### MoleculeNet Benchmarking of ChemXTree

To comprehensively evaluate the ChemXTree, we utilized the MoleculeNet databases developed by Wu et al.^40^ ChemXTree is mainly developed for single-target classification. Due to ChemXTree’s utilization of decision tree algorithms, transforming multitask classification problems into multiple binary classification tasks is a common approach. However, this transformation leads to increased computational costs and deployment difficulties. Considering our limited computational resources, we did not conduct tests on tasks involving multitask binary classifications. In this evaluation part, we adhered to the data processing and evaluation methods presented in previous work. After obtaining the optimal hyperparameters through Bayesian optimization and running the model three times, we conducted a comparison with various existing models.

**Figure 2:**
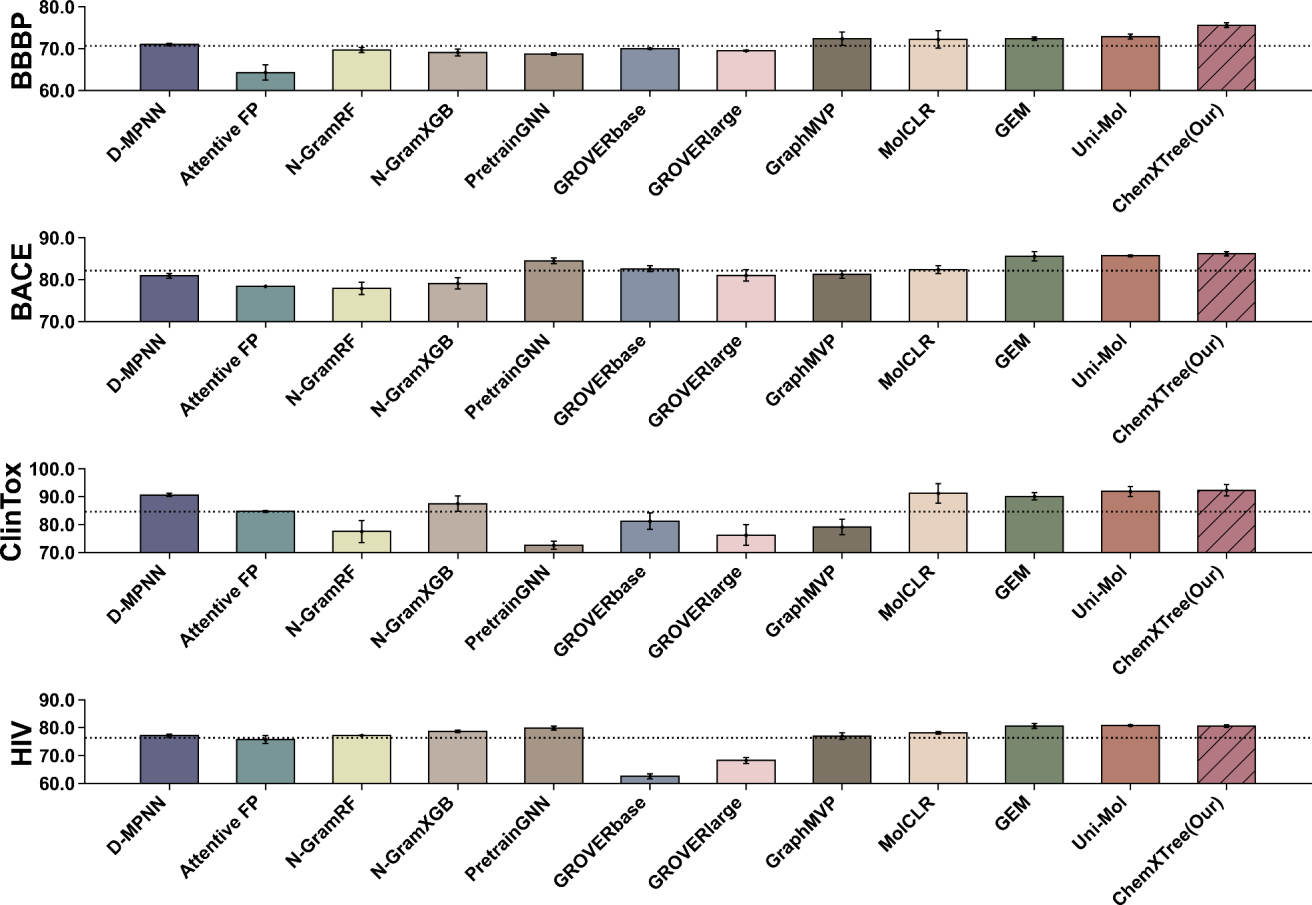
Performance of ChemXTree on MoleculeNet Classification Tasks. This chart compares the ROC-AUC scores of ChemXTree and other models on various MoleculeNet classification tasks, including BBBP, BACE, ClinTox, and HIV data sets. The horizontal axis denotes the models, while the vertical axis represents their ROC-AUC scores (%).

As shown in Figure 2 and Table 1, ChemXTree performs comparably or slightly better than the SOTA models in all the data sets. Notably, ChemXTree excels in predicting tasks such as BBBP, BACE, and ClinTox. A potential factor could be ChemXTree’s strategic feature selection combined with its tree-based output layer, offering an equilibrium between fitting the data and generalizing well, especially with small data sets. Specifically, compared to D-MPNN^41^ where similar molecular feature extraction is applied, ChemXTree demonstrates a consistent trend of improvement across all data sets, boosting the ROC-AUC by 6% for both BBBP and BACE tasks. This indicates that combining tree-based models with other approaches can enhance the utilization of feature information and significantly boost the performance of the GNN model. Moreover, models such as N-GramRF,^33^ N-GramXGB, PretrainGNN,^45^ GROVERlarge,^46^ GROVERbase, MolCLR,^19^ and Uni-Mol^47^ undergo pretraining and fine-tuning process. In contrast, ChemXTree attains comparable results without this step, indicating the potential of its structure and algorithms even in the absence of pretraining.

**Table 1:** Performance of ChemXTree and Other Models Across BBBP, BACE, HIV, and ClinTox Datasets with Best ROC-AUC Score Denoted in Bold (Values in Parentheses Represent Standard Deviation)

Data sets	BBBP	BACE	HIV	ClinTox
D-MPNN^41^	71.0 (0.3)	80.9 (0.6)	77.1 (0.5)	90.6 (0.6)
Attentive FP^48^	64.3 (1.8)	78.4 (0.02)	75.7 (1.4)	84.7 (0.3)
N-GramRF^33^	69.7 (0.6)	77.9 (1.5)	77.2 (0.1)	77.5 (4.0)
N-GramXGB^33^	69.1 (0.8)	79.1 (1.3)	78.7 (0.4)	87.5 (2.7)
PretrainGNN^45^	68.7 (1.3)	84.5 (0.7)	79.9 (0.7)	72.6 (1.5)
GROVERbase^46^	70.0 (0.1)	82.6 (0.7)	62.5 (0.9)	81.2 (3.0)
GROVERlarge^46^	69.5 (0.1)	81.0 (1.4)	68.2 (1.1)	76.2 (3.7)
GraphMVP^49^	72.4 (1.6)	81.2 (0.9)	77.0 (1.2)	79.1 (2.8)
MolCLR^19^	72.2 (2.1)	82.4 (0.9)	78.1 (0.5)	91.2 (3.5)
GEM^50^	72.4 (0.4)	85.6 (1.1)	80.6 (0.9)	90.1 (1.3)
Uni-Mol^47^	72.9 (0.6)	85.7 (0.2)	**80. 8** (0.3)	91.9 (1.8)
ChemXTree(Our)	**75. 6** (0.6)	**86. 2** (0.5)	80.6 (0.5)	**92. 3** (0.8)

### Comprehensive Performance Evaluation of ChemXTree Across Diverse Drug Data Sets

For a comprehensive benchmark, we tested ChemXTree on 8 additional drug data sets, including AMES, CYP2C9_Substrate, CYP2D6_Substrate, CYP3A4_Substrate, CYP2C9_inhibitor, CYP2D6_inhibitor, CYP3A4_inhibitor, and Bioavailability. These data sets have been widely adopted in previous works as benchmarks.^51^ Specifically, our data set selection considers AMES, CYP2C9_inhibitor, CYP2D6_inhibitor, and CYP3A4_inhibitor as large-scale data sets, and CYP2C9_Substrate, CYP2D6_Substrate, CYP3A4_Substrate, and Bioavailability as small-scale data sets, in order to cover diverse data characteristics and model capabilities. We compare our ChemXTree against 10 SOTA methods, spanning from conventional machine learning algorithms, e.g., XGBoost to emerging deep graph neural networks including DMPNN, AttentiveFP, Graph Attention Network (GAT), etc., as well as pretrained models like Uni-Mol and GROVER (GROVERbase is chosen as the representative of GROVER due to their performance in MoleculeNet). The detailed benchmark model information could be found in Materials and Methods. For comparison, we optimize hyperparameters and follow the same data set splitting and evaluation metrics (ROC-AUC) as MoleculeNet recommend.

**Figure 3:**
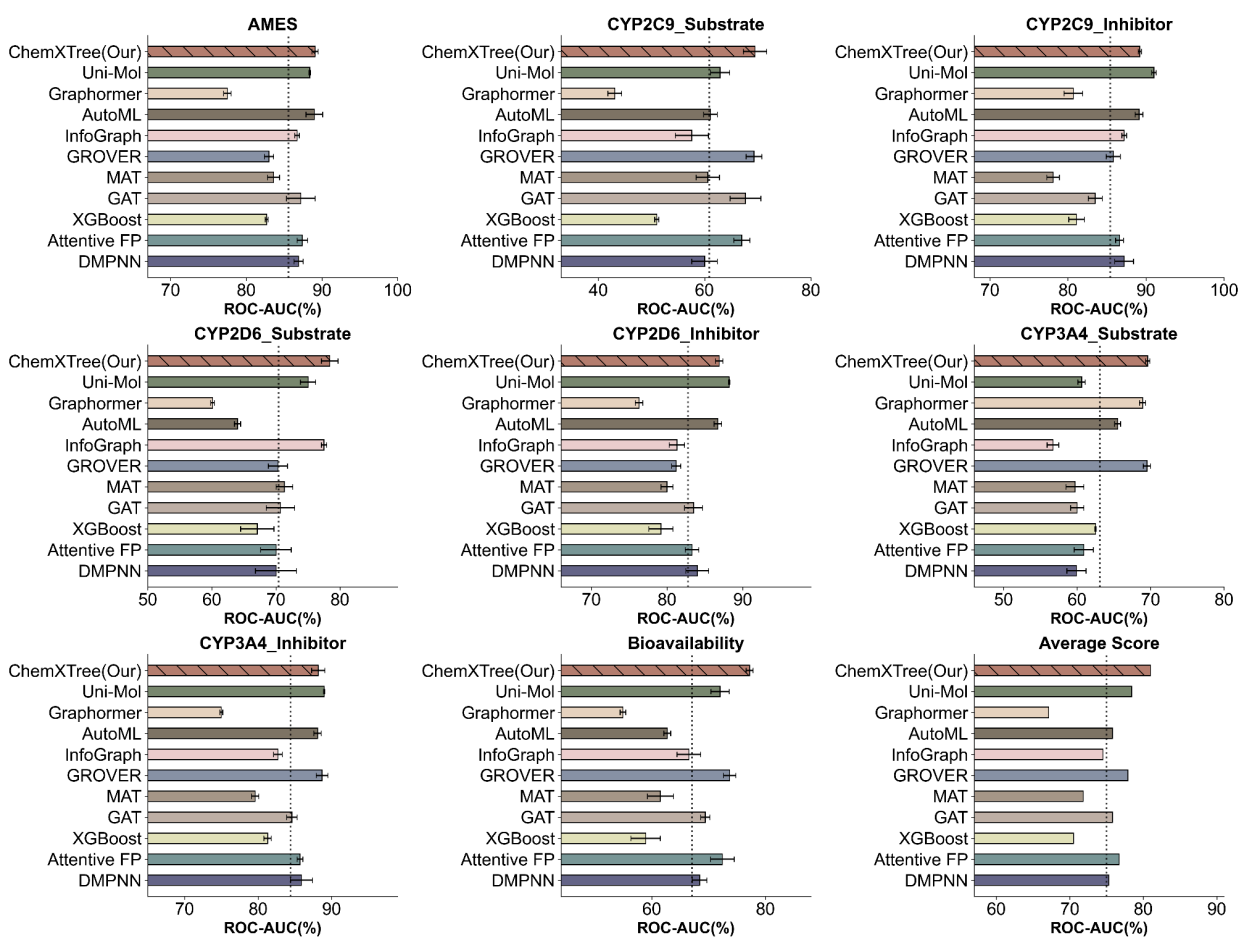
This comprehensive chart showcases the performance comparison of ChemXTree against various baseline models across a diverse range of drug benchmark data sets. The data sets encompass AMES, CYP2C9_Substrate, CYP2C9_Inhibitor, CYP2D6_Substrate, CYP2D6_Inhibitor, CYP3A4_Substrate, CYP3A4_Inhibitor, and Bioavailability. The horizontal axis represents the ROC-AUC percentage, while the vertical axis denotes the model names.

As shown in Figure 3, our ChemXTree achieves superior average ROC-AUC scores across all 8 data sets compared to other methods, demonstrating its strong generalization capability. Specifically, ChemXTree attains the best ROC-AUC performance on the AMES, CYP2C9, CYP2D6, CYP3A4, and bioavailability data sets, with 89.1%, 69.5%, 78.4%, 69.6%, and 77.2% respectively. For the three larger data sets of CYP2C9 Inhibitor, CYP2D6 Inhibitor, and CYP3A4 Inhibitor, ChemXTree ranks second, only behind the pretrained Uni-Mol model and on par with GROVER. This is reasonable since Uni-Mol benefits from pretraining on external millions of molecular data, granting better generalization to represent unseen cases. In contrast, ChemXTree learns representations from scratch solely based on the training data yet still surpasses other methods without pretraining, verifying its modeling effectiveness. Overall, ChemXTree achieves an average ROC-AUC of 81.0% across the eight data sets, significantly outperforming all other methods and demonstrating its superiority and competitiveness. In summary, the benchmark experiments thoroughly prove ChemXTree’s superiority and competitiveness in molecular property prediction tasks on both small- and large-scale data sets.

### Ablation and Substitution Analysis in ChemXTree

To analyze ChemXTree’s enhancements, we took the molecular encoding from the top-performing ChemXTree on BACE/BBBP/HIV data sets to conduct the ablation experiments by swapping its prediction output with a basic FFN and XGBoost. The basic FFN configuration was from Chemprop’s default set and it was trained over 1000 epochs.^52^ We conducted a grid search over the following hyperparameters: batch_sizes of {16, 32, 64} and learning_rates of {0.001, 0.002, 0.004}. For the XGBoost, we employed Bayesian optimization within a defined parameter space: learning_rate between 0.004 and 1.0, max_depth in the interval [4, 20], and n_estimators spanning [20, 400]. We incremented the Bayesian search iterations, starting from 30 and gradually increasing to 50, 200, and 300. Besides, the lambda and alpha, which are regularization terms, are in the range of [0,10]. For these two layer substitutions, we recorded the best performance model for the comprehensive analysis, respectively.

**Figure 4:**
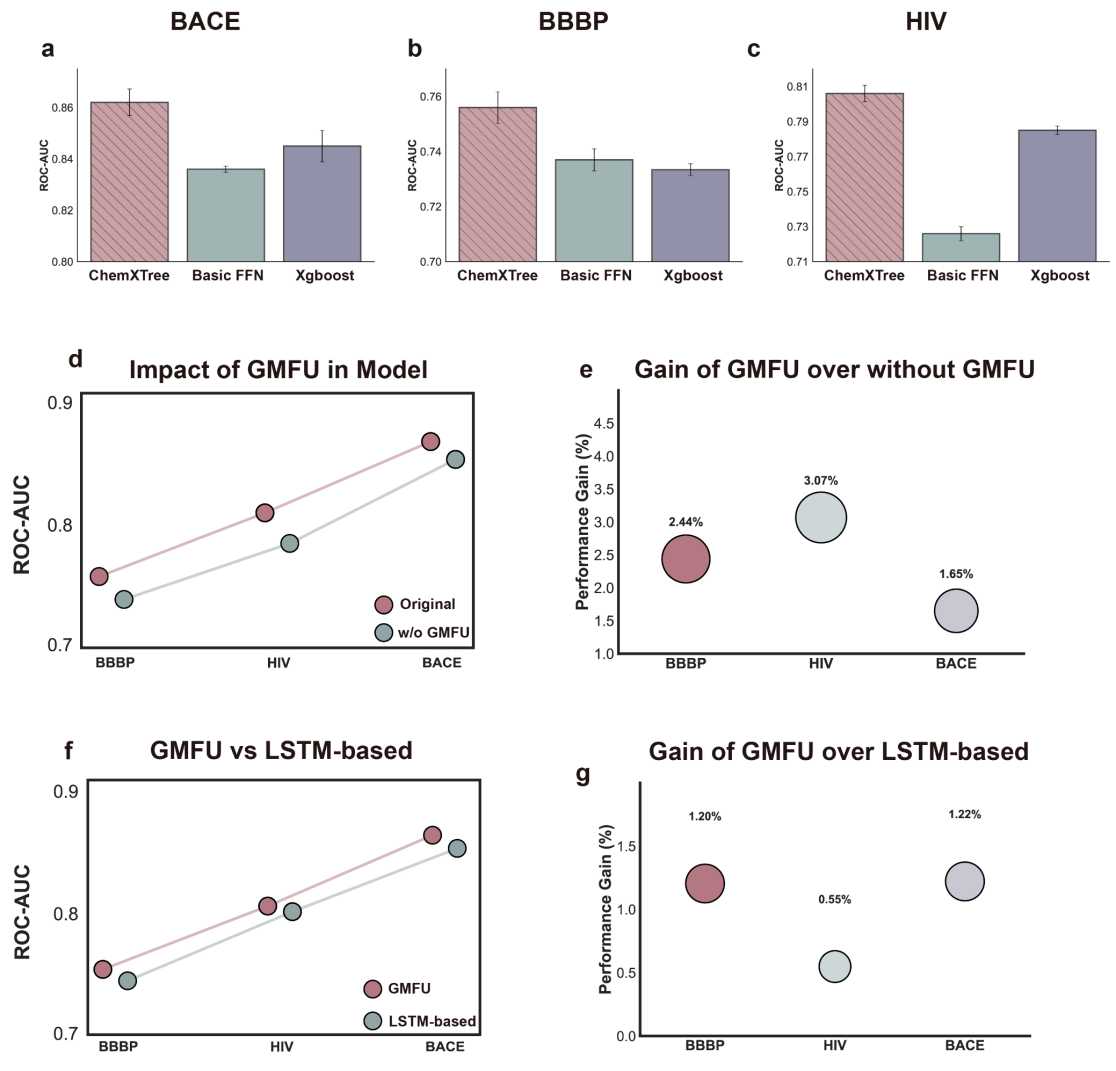
(a–c) Performance comparison of ChemXTree with Basic FFN and XGBoost output layers across the BACE, BBBP, and HIV data sets, with all models using the same molecular encoding. (d) Performance comparison of ChemXTree with and without GMFU module across BACE, BBBP, and HIV data sets. (e) Improvement in performance achieved by the GMFU module across BACE, BBBP, and HIV data sets. (f) Performance comparison of ChemXTree with GMFU module and LSTM-based module across BACE, BBBP, and HIV data sets. (g) Performance advantage of the GMFU module compared to the LSTM-based variant for BACE, BBBP, and HIV data sets.

In Figure 4a–c, a comparison of three output layers using identical encoded inputs shows that ChemXTree outperforms both basic FFN and XGBoost in terms of classification efficacy. We computed the average ROC-AUC score of each output layers across three data sets, with the performance scores ranking as follows: ChemXTree > XGBoost > basic FFN. Specifically for the HIV data set (Figure 2C), which has a significant class imbalance with approximately 1:27 ratio of positives to negatives, tree-based approaches clearly outperform basic FFN. This can be attributed to the tree methods’ branching mechanisms, which are highly effective at capturing nonlinear relationships within the data. Besides, while XGBoost does not surpass the basic FFN on the BBBP data set, its respective performance in other cases supports its viability as an effective substitute for the basic FFN layer in a range of computational scenarios.

To assess the effectiveness of the GMFU module in ChemXTree, we also conducted an ablation study to evaluate the effectiveness of GMFU. Employing identical inputs and optimal performance parameters, we compared the original ChemXTree model with variants lacking GMFU using identical inputs and optimal performance parameters across three data sets. In Figure 4d presented, “w/o GMFU” denotes the ChemXTree configuration excluding the GMFU module. The results revealed a decline in model performance for all three data sets when GMFU was removed. Figure 4e further illustrates the importance of the GMFU component for optimizing ChemXTree’s performance: on the BBBP data set, GMFU provided a 2.44% improvement, while in the HIV and BACE data sets, it yielded gains of 3.07% and 1.65%, respectively. These results indicate that GMFU is an essential part of optimizing the ChemXTree model, as it enhances the feature selection function and improves the model’s performance.

In addition to the GMFU, we explored a variant inspired by long short-term memory (LSTM) architecture.^53^ To distinguish between these modules by mechanism, we refer to them as GMFU and LSTM-based, respectively. It is noteworthy that the model adaptation for the LSTM-based variant is nearly identical to the treatment of GMFU. As depicted in Figure 4f,g, the original ChemXTree outperforms its LSTM-based variant on all three data sets, achieving an average performance boost of 1%. In contrast to the LSTM-based approach, which employs a complex three-gate architecture for feature selection, the GMFU strategy adopts a more efficient two-gate design consisting solely of reset and update gates. This streamlined structure not only reduces the number of model parameters but also leads to a more memory-efficient architecture. As evidenced in Table S2, GMFU resulted in faster average training times compared with the LSTM-based variant.

### Comparative Visualization of Different Molecular Representations in CYP2D6_Substrate Data Set

**Figure 5:**
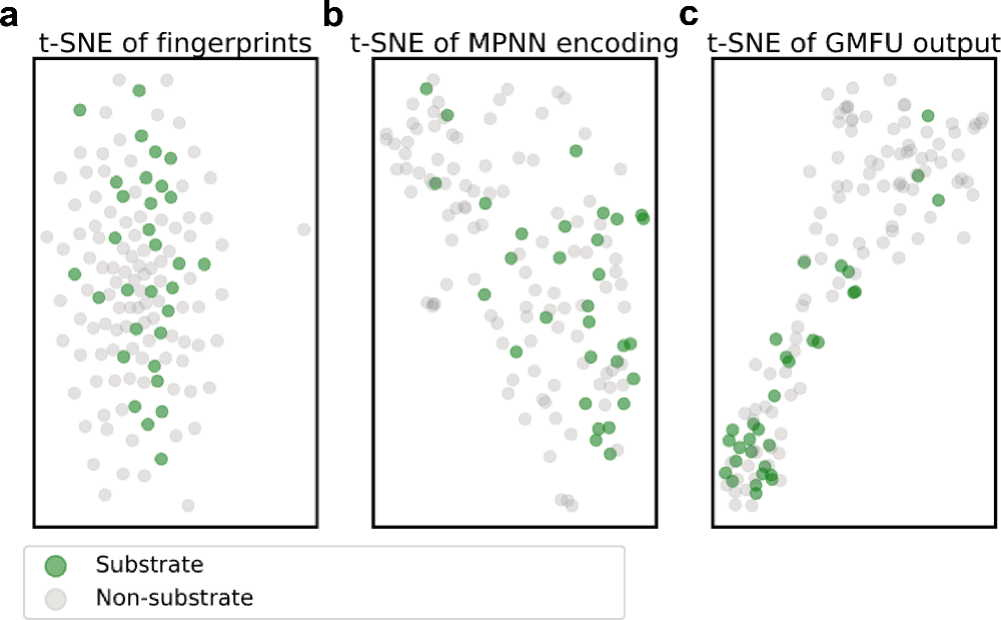
t-SNE visualization of CYP2D6_Substrate testset, showing 2048-bits Morgan Fingerprints, MPNN-encoded Embeddings, and ChemXTree hidden layer Representations. The plots in t-SNE were generated where ground truth labels 1 (substrate) and 0 (nonsubstrate) were denoted in green and gray, respectively.

We use CYP2D6_Substrate testset for t-SNE analysis to visually compare three different representations: 2048-bit molecular Morgan fingerprints,^54^ MPNN-encoded embeddings, and hidden layer representations from the ChemXTree GMFU module before feeding into neural decision tree^55^ (Figure 5a–c). The t-SNE results revealed dispersed distributions for the Morgan fingerprints, indicating that Morgan fingerprints may not effectively capture the underlying chemical properties relevant to CYP2D6 substrate binding. In contrast, the MPNN embeddings showed some subtle clustering after t-SNE dimensionality reduction. Notably, the GMFU module representations displayed considerably tighter clustering after t-SNE dimensionality reduction. This suggests a superior ability to distinguish between molecules with varying efficacy in the high-dimensional space of CYP2D6 substrate binding.

## Conclusion

In this work, we have proposed ChemXTree, a feature-enhanced GNN-NDT Framework for molecular property prediction in drug discovery. We comprehensively evaluated ChemXTree on both the MoleculeNet benchmark and an additional eight drug discovery data sets. The results demonstrated SOTA performance, with ChemXTree outperforming current top models on 8 out of the 12 data sets. Ablation studies and model substitution experiments further validated the effectiveness of the GMFU and neural decision tree modules, highlighting their contributions to feature selection and classification. t-SNE analysis on the CYP2D6 substrate data set indicated that the GMFU module significantly enhanced the separation between substrates and nonsubstrates, indicating its ability to capture key pharmacophoric features.

In the ChemXTree, we adopt a simple representation method based on MPNN to highlight the significance of the nonrepresentation component in ChemXTree. Therefore, to address the current limitations and elevate the application to a higher level, our future work will focus on GNN pretraining representation, feature extraction, and balancing input complexity with computational requirements. Additionally, simplifying the workflow and reducing the number of parameters are also key directions for optimization. In summary, ChemXTree presents a competitive and innovative end-to-end graph representation learning framework for drug discovery, particularly excelling in small sample learning scenarios. ChemXTree holds great promise to accelerate the identification of novel drug candidates and contribute to the advancement of computational drug discovery.

## Materials and Methods

Message Passing Neural Networks (MPNNs) efficiently capture the molecular graph structures of small organic molecules through nodes and edges. The stochastic nature of their training renders the outputs highly sensitive to hyperparameter settings and variability in training. Although MPNNs offer linear features as an alternative to conventional vertex and edge representations, reliably determining the optimal output from multiple training iterations and evaluating the significance of specific features for particular tasks continue to be challenging.

Decision trees are widely recognized for their capability to partition a feature space into distinct and nonoverlapping regions, based on specific feature values. In statistical learning context, for a given feature’s random variable *X*, the decision tree applies a set of “if-then” rules based on conditional probabilities *P*(*Y*|*X*), showcasing the classification outcomes of these rules. These rules facilitate a mutually exclusive and complete classification of instances into positive or negative categories. In the context of drug discovery, the effectiveness to differentiate between classes is crucial. Hence, binary decision tree models offer an inherent efficiency in modeling binary-class drug molecule data sets.

Ideally, a training data set contains a minimal subset including all necessary “if-then” rules for classification. The goal for a classifier is to accurately identify instances within this subset. In the context of decision trees, Information Gain (IG) is commonly employed as a criterion for feature selection and tree pruning, identifying this minimal subset. However, IG’s deterministic nature, depending on data set composition, raises the risk of overfitting in high-dimensional spaces, such as molecular fingerprints. It exposes the limitations of decision trees in adapting loss dynamically compared to deep learning models with backpropagation machanism.

The Gate Recurrent Unit (GRU) stands out in Natural Language Processing (NLP) models for its gating mechanisms, which simplifies configuration and boosts denoising performance with fair computational demands. However, molecular representations typically lack temporal, hierarchical, and sequential characteristics and often show sparsity and uniformity.

Building on these insights, we designed ChemXTree, which integrates a novel fingerprint strategy based on MPNN with a data augmentation architecture, Gate Modulation Feature Unit (GMFU). This new fingerprint representation offers remarkable robustness, while GMFU merges the strengths of boosting trees and gated mechanisms, further enhanced by gradient back-propagation, to improve adaptability and control in molecular property prediction.

### Data Sets

MoleculeNet includes 16 data sets covering various scientific domains like Quantum Mechanics, Physical Chemistry, and Biophysics. These data sets are utilized for benchmarking machine learning models in the respective fields. Among the 16 data sets, there are several binary classification tasks in our comparison:

(1) BBBP: The Bloodbrain barrier penetration (BBBP) data set. BBBP data set evaluates the permeability of small molecules across the blood-brain barrier. It comprises 2039 validated molecules, featuring a positive-to-negative sample ratio of approximately 3.2:1.

(2) BACE: The BACE data set provides a comprehensive array of both quantitative (IC50 values) and qualitative (binary classification labels) data, evaluating the binding efficacy of inhibitors aimed at human β-secretase 1 (BACE-1). The data set comprises 1513 molecules, with an inactive-to-active label ratio of approximately 1.23:1.

(3) HIV: The HIV data set originates from the Drug Therapeutics Program’s AIDS Antiviral Screen. It contains data on 41,127 molecules and their efficacy in inhibiting HIV replication. The data set features a ratio of inactive to active molecules of approximately 27.5:1.

(4) ClinTox: The ClinTox data set provides a comparative analysis between FDA-approved drugs and molecules that failed in clinical trials due to toxicity concerns. It includes 1478 molecules, with ratios of FDA-approved to unapproved drugs at approximately 14.9:1 and clinical toxicity-positive to -negative molecules at approximately 12.2:1.

For benchmarking on additional 8 ADMET data sets. Our ADMET data sets are derived from previous works.^5,51^ We thank the Therapeutics Data Commons for providing access to these valuable resources.

Ames Mutagenicity (Toxicity): The Ames Mutagenicity data set focuses on the potential mutagenic effects of molecules using the Ames test, which identifies substances that may damage DNA.^56^ In this work, we refer to the Ames Mutagenicity data set as AMES.

CYP2C9, CYP2D6, and CYP3A4 Substrate (Metabolism): These data sets examine the roles of CYP450 enzymes 2C9, 2D6, and 3A4 in metabolizing endogenous and foreign molecules. The tasks of these data sets are predicting whether molecules act as substrates for these enzymes.^57^

CYP2C9, CYP2D6, and CYP3A4 inhibitor (Metabolism): In contrast to the Substrate data sets, these data sets primarily aim to predict whether molecules inhibit the metabolic activity of CYP450 enzymes 2C9, 2D6, and 3A4.^58^

Bioavailability (Absorption): This data set represents the rate and extent at which active molecules are absorbed from a drug product and become effective at the site of action. The data set is used for predicting the activity of bioavailability.^59^

### Spilt and Evaluation Metric

Scaffold splitting is commonly adopted in cheminformatics as a way to better evaluate the model generalization. While random splitting offers simplicity in implementation, it often inadequately represents the model’s ability to generalize to novel data. Scaffold splitting, on the other hand, aims to divide the data set into subsets based on structurally distinct molecules. This approach poses a greater challenge to learning algorithms compared with random splitting, thereby providing a more robust measure of generalization.

In the case of data sets from MolecuNet, we follow a previously established 8:1:1 ratio for splitting the data into training sets, validation sets, and test sets. For data sets that are not part of MoleculeNet, we employ a 7:1:2 scaffold spliting. To establish a unified standard, we adhere to the guidelines set by MoleculeNet and other previous works, using ROC-AUC as the evaluation metric across all our experiments. This allowed direct comparison to existing benchmarks and studies. Adhering to MoleculeNet’s criteria, PRC-AUC is employed for data sets with a positive sample rate below 2%; otherwise, ROC-AUC is preferred. Therefore, we use ROC-AUC for evaluation in these data sets.

### Comparison Models

N-Gram: N-Gram model introduced by Liu et al. offers a simple, unsupervised approach to represent molecules by embedding vertices and assembling them in short walks within the graph.^33^

MolCLR: MolCLR proposed by Wang et al. is a framework for molecular representation learning that applies contrastive learning to encode molecular structures to potentially capture the underlying patterns and relationships in molecular data.^19^

GraphMVP (Graph Multiview Pretraining): GraphMVP developed by by Liu et al. represents a pretraining method for graph neural networks, leveraging self-supervised learning to exploit the relationships and consistencies between 2D topological structures and 3D geometric views.^49^

PretrainGNN: PretrainGNN is a new strategy developed by Hu et al. for pretraining Graph Neural Networks (GNNs) that simultaneously trains on individual nodes and entire graphs, enhancing both local and global representations.^45^

GEM (Geometry-enhanced Molecular representation): GEM improves molecular property prediction by integrating molecular geometry into a Graph Neural Network (GNN). It utilizes a specialized GeoGNN architecture to simultaneously consider the influence of atoms, bonds, and bond angles, thereby crafting a more detailed representation of molecules.^50^

AttentionFP: AttentionFP is a model proposed by Xiong et al. This model employs a graph attention mechanism to focus on the most crucial parts of the molecular structure, particularly the nonlocal intramolecular interactions.^48^

GAT (Graph Attention Networks): GAT is a class of graph neural networks distinguished by their utilization of attention mechanisms enabling GATs to selectively prioritize and integrate information from adjacent nodes.^60^

MAT (Molecular Attention Transformer): MAT developed by Maziarka et al. is based on the Transformer designed for molecular representation. It enhances the self-attention mechanisms of the Transformer by incorporating interatomic distances and molecular graph structures, allowing for a deeper understanding of molecular features.^61^

GROVER: This is a pretrained model designed by Rong et al. employing a self-supervised message passing transformer architecture, integrating message passing networks with a transformer framework to effectively learn molecular representations from unlabeled data. GROVER has two sizes: GROVERbase and GROVERlarge with different hidden layers.^46^

InfoGraph: InfoGraph is an graph representation learning method proposed by Sun et al. It maximizes mutual information between graph-level and substructure representations, enabling robust graph-level learning.^62^

Automated Machine Learning (AutoML): AutoML denotes the process automation for selecting, fine-tuning, and training machine learning models. In this work, the input is sourced from a 2048-bit Morgan Fingerprints, followed by the automatic fine-tuning of a Feedforward Neural Network (FFN).^63^

Graphormer: Graphormer is a novel architecture for molecular property prediction proposed by Ying et al. in 2021. It combines graph neural networks (GNNs) with the Transformer structure widely used in natural language processing.^64^

Uni-Mol: Zhou et al. have developed Uni-Mol, a 3D molecular representation learning framework that incorporates a comprehensively pretrained model, adept at processing a broad spectrum of molecular and protein pocket data for diverse molecular representation tasks.^47^

Among these models, N-gram, PretrainGNN, GROVER, GraphMVP, MolCLR, GEM, and Uni-Mol are pretrained model and others are designed without pretrained (namely, fine-tuned model).

### Input and Representation

To make the representation simpler, we applied MPNN to encode molecule graphs, which includes vertice and edges infomation, into numerical linear embeddings. For binary classification tasks, we have an input data set  mapping from feature space  to the output space ,
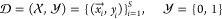
1whereas the *i*th molecule’s representation vector  is a *d*-featured array corresponding to a target label . Initialization involves setting model parameters to reflect the average target value from the training data.

### Message Passing Neural Networks Module

Our Message Passing Neural Networks (MPNN) Module is developed from the Chemprop framework and has undergone fine-tuning. To be more specific, as with many existing works on molecular graph representation, the initial required input is canonical SMILES without atom mapping. In our MPNN module, atoms and bonds of the molecules correspond to vertices V and edges E in the graph structure, respectively. We encode the features of the atoms (atomic numbers, numbers of bonds, formal charges, chirality, quantity of hydrogen, hybridization, aromaticity of the atom, and atomic mass) and the features of the bonds (types, in conjugation/ring, stereochemical information) to obtain the initial vectors. Therefore we can get
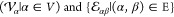


Furthermore, the initial directed edge features are obtained by connecting the atomic features of the first atom of the bond to the corresponding undirected bond feature . Here, ϕ() is a simple function of concatenation.
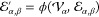
Subsequently, the initialized features are passed into the MPNN. Herein, the edge features are first processed through a simple network layer endowed with a ReLU activation function and learnable weights *W*_*i*_, where . In our model, the values *h*^0^_α, β_ = 300 and  are taken. Therefore:
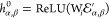


The directed edge attributes are subsequently updated through three iterative rounds of message passing, based on the local topology:
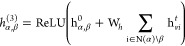
where  and 

Let us denote
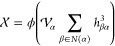
With this definition, the final hidden states can be expressed, which are then aggregated to form the atomic embeddings, as follows:
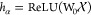
Therefore, the complete atomic embedding can be represented as
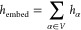
Notably, we apply a bagging strategy to MPNN-generated fingerprints, each of size 5, to decrease the error in the graph-to-vector transformation, thereby emphasizing similar features while still preserving less prominent ones. This approach ensures that subsequent learners have the opportunity to evaluate the importance of these less focused features.

### Gate Modulation Feature Unit

The attention component in the Gate Modulation Feature Unit (GMFU) operates as follows:
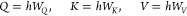
2where , *F* is the dimension of input features, and *d* is the dimension for each attention head. *Q*, *K*, and *V* are split along the last dimension *d* into *n*_heads_ different heads.
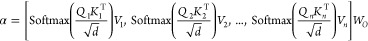
where, .

After application of the self-attention mechanism, the feature vectors are passed into a gate control mechanism that plays a crucial role in the process of feature selection. This gate control mechanism has been adapted from GRU and GANDALF.

Inspired by previous works that have effectively utilized sparsity in neural networks, this work also adopts the t-softmax activation function proposed by Baazy et al. in 2023 for feature masking. Specifically, the mask *M*_*n*_ is generated using the t-softmax function applied to a learnable parameter vector *F*_*n*_ and a tuning parameter *t*, as shown in the equation *M*_*n*_ = t-softmax(*F*_*n*_, *t*). This mask is then used to perform an element-wise multiplication with the input features *X*, resulting in the masked features δ_*t*_ as described in the equation δ_*t*_ = *M*_*n*_ ⊙ *X*. Also, priors are introduced by initializing the masks using Beta Distribution: *F*_*n*_ ∼ Beta(α_*n*_, β_*n*_), where α_*n*_ and β_*n*_ are drawn from uniform distributions: α_*n*_ ∼ Uniform(0.5, 10), β_*n*_ ∼ Uniform(0.5, 10).

Similar to the standard GRU and GANDALF, we set the corresponding reset gate *r*_*t*_ and update gate *z*_*t*_ as follows:
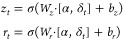
σ is the sigmoid activation function, which ensures the output is in the range of [0,1]. With these gates, we can further specify the hidden state *H*_*t*_ and the candidate feature *H̃*_*t*_. They are defined as
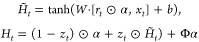
where Φ is a parameter in the range of [0,1]. The default value of Φ in the model is 0.05.

### Differentiable Neural Decision Tree

We input the features processed through GMFU into a differentiable binary decision tree. For such a decision tree, its role is to transform a *k*-dimensional input into a 2D-dimensional output. Although traditional decision trees have advantages like ease of deployment and straightforward algorithms, their methods are nondifferentiable. Therefore, definite soft decision binary trees are considered in this work.

Inspired by prior research,^44,65^ the Soft Binning Function *g* and the decision stump *o*_*i*_ in this work serve as specialized counterparts to the splitting criterion and the decision node found in traditional decision trees, respectively.
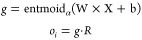
where 

Definite decision binary trees in the model utilize all available features for each split through a linear combination of nonlinear functions. A learnable feature mask  is introduced to efficiently combine the outputs *o*_*i*_, allowing for scalability and comprehensive feature consideration.
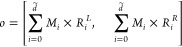
where d̃ is the dimensions of the learned feature representation from GFLUs and *D* is the depth of the tree.

In the Probably Approximately Correct (PAC) learning framework, if a differentiable binary decision tree of a fixed depth (i.e., within a polynomial time complexity) can evidently perform better than random guessing, then it is qualified as an effective learner. To further enhance this, it is feasible to rationally select a boosting algorithm tailored to this context.^66^ Inspired by this theorem, we boosted our finite neural decision tree with specific tricks. Unlike classical boosting techniques, we chain the trees sequentially, with each tree’s output feeding into the next.^44,67,68^ Formally the output for the *i*th tree follows
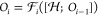
where ,  and [;] represents a concatenation operation.

Denoting the previously described tree as *t*_*i*_, the boosting tree ensemble is expressed as
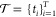
Hence, the set of leaf responses  from  is
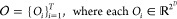
After scaling with multihead attention, where , we obtain the leaf responses ,
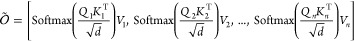
3passes through *T* distinct output linear layers, corresponding to each tree, resulting in the desired output vector. Then we denote the set of linearly transformed outputs by , where
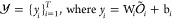
and for each tree, *W*_*i*_ and *b*_*i*_ are learnable parameters which transforms the leaf response vector into the desired output. The final prediction is formulated as an estimator of the targets, represented by
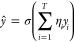
4

### Training

The ChemXTree and baseline models were trained on the NYU Greene and NYUAD Jubail high-performance computing (HPC) clusters. Specifically, we utilized NVIDIA’s A100/H100 GPUs for our experiments in most small data sets. For large size data sets, we escalated our computational capacity by deploying dual GPUs. In every experiment conducted, drawing inspiration from the parameter tuning practices customary in traditional machine learning, we harnessed the prowess of Bayesian optimization. This method was utilized to meticulously traverse the parameter space comprising the learning rate, batch size, tree depth, tree breadth, the count of heads in the multihead attention mechanism within GMFU, and the quantity of GRUs, alongside the dropout rate. This meticulous exploration was performed through 100 Bayesian searches in each experiment. In the ablation experiment and comparative experiment in ADMET data sets, we fine-tuned key parameters like learning rate, epoch, and batch size for each model. For the detailed hyperparameters, please see the Supporting Information.

All the benchmark data can be found in the https://moleculenet.org/ and https://tdcommons.ai/. Also, the code and the data sets from section 2.6 for the models and results can be found in the codeocean platform https://codeocean.com/capsule/2818241/tree.

## Supporting Information Available

Comparative analysis of ROC-AUC scores, dataset summary, analysis of feature importance, parameter specification, number of model parameters, and model optimization (PDF)
