# Supplementary material for: ChemXTree: A Feature-Enhanced Graph Neural Network-Neural Decision Tree Framework for ADMET Prediction
Source: J Chem Inf Model. 2024 Nov 5;64(22):8440–52. doi: 10.1021/acs.jcim.4c01186 (PMC11600499; doi:10.1021/acs.jcim.4c01186)
Supplement: Supplementary file 1 — ci4c01186_si_001.pdf [file ci4c01186_si_001.pdf]

ChemXTree: A Feature-Enhanced Graph Neural Network-Neural Decision Tree Framework for ADMET Prediction

Yuzhi Xu,[†,‡,△] Xinxin Liu,[¶,§,△] Wei Xia,[†,‡,△] Jiankai Ge,[‖] Cheng-Wei Ju,[⊥] Haiping Zhang,[#] and John Z.H. Zhang[*,†,‡,#,@]

†Shanghai Frontiers Science Center of Artificial Intelligence and Deep Learning and NYU-ECNU Center for Computational Chemistry, NYU Shanghai, Shanghai 200062, China

‡Department of Chemistry, New York University, New York, New York 10003, United States

¶Department of Computer and Information Science, University of Pennsylvania, Philadelphia, Pennsylvania 19104, United States

§Department of Materials Science and Engineering, University of Pennsylvania, Philadelphia, Pennsylvania 19104, United States

‖Chemical and Biomolecular Engineering, University of Illinois at Urbana-Champaign, Urbana, Illinois 61801, United States

⊥Pritzker School of Molecular Engineering, The University of Chicago, Chicago, Illinois 60615, United States

#Faculty of Synthetic Biology and Institute of Synthetic Biology, Shenzhen Institute of Advanced Technology, Shenzhen 518055, China

@Shanghai Engineering Research Center of Molecular Therapeutics and New Drug Development, School of Chemistry and Molecular Engineering, East China Normal University, 200062, China

△These authors contributed equally to this work

E-mail: john.zhang@nyu.edu

Performance Evaluation

Downstream Dataset

Table S1: Comparative Analysis of ROC-AUC Scores Across Eight Drug Datasets for ChemXTree and Other Leading Models in AMES, CYP Enzyme Substrate and Inhibitor, and Bioavailability, with Top Performances in Bold and Second Best Underlined (Values in Parentheses Represent Standard Deviation)

Datasets	AMES	CYP2C9 Substrate	CYP2C9 Inhibitor	CYP2D6 Substrate	CYP2D6 Inhibitor	CYP3A4 Substrate	CYP3A4 Inhibitor	Bioav	**Average**
DMPNN	86.9(0.6)	60.0(2.4)	87.2(1.2)	70.0(3.2)	84.0(1.5)	59.9(1.3)	85.9(1.5)	68.4(1.3)	75.3
Attentive FP [1]	87.4(0.7)	67.0(1.5)	86.6(0.5)	70.0(2.4)	83.3(0.9)	60.9(1.3)	85.7(0.4)	72.4(2.1)	76.7
XGBoost	82.7(0.2)	51.0(0.4)	81.1(1.0)	67.1(2.6)	79.2(1.6)	62.5(0.1)	81.3(0.5)	58.9(2.6)	70.5
GAT [2]	87.2(1.9)	67.7(2.9)	83.5(0.9)	70.7(2.2)	83.5(1.2)	60.0(0.9)	84.6(0.7)	69.4(0.8)	75.8
MAT [3]	83.6(0.8)	60.6(2.2)	78.1(0.8)	71.3(1.3)	80.0(0.8)	59.7(1.2)	79.6(0.5)	61.5(2.3)	71.8
GROVER	83.0(0.6)	69.3(1.5)	85.8(0.9)	70.3(1.5)	81.2(0.6)	69.5(0.5)	88.7(0.8)	73.7(1.1)	77.9
InfoGraph [4]	86.7(0.3)	57.6(3.1)	87.2(0.3)	77.5(0.4)	81.3(1.0)	56.7(0.8)	82.7(0.6)	66.5(2.1)	74.5
AutoML	89.0(1.1)	61.1(1.3)	89.1(0.5)	64.0(0.5)	86.7(0.5)	65.5(0.4)	88.1(0.5)	62.7(0.6)	75.8
Graphormer [5]	77.5(0.5)	43.1(1.3)	80.7(1.2)	60.1(0.3)	76.3(0.5)	68.9(0.4)	75.0(0.2)	54.9(0.5)	67.1
Uni-Mol	88.4(0.1)	62.9(1.8)	**91.0(0.3)**	75.0(1.2)	**88.2(0.1)**	60.6(0.5)	**89.0(0.1)**	72.0(1.6)	78.4
ChemXTree(our)	**89.1(0.4)**	**69.5(2.2)**	89.2(0.2)	**78.4(1.3)**	86.9(0.5)	**69.6(0.3)**	88.2(0.9)	**77.2(0.6)**	**81.0**

Datasets Summary

Table S2: Dataset summary: size, biochemical field, and task type.

Dataset	Data size	Dataset Field	Task Type
BBBP	2039	Permeability	Classification
BACE	1513	Inhibitor	Classification
HIV	41127	Antiviral	Classification
ClinTox	1478	Toxicity	Classification
AMES	7255	Toxicity	Classification
CYP2C9 Substrate	666	Metabolism	Classification
CYP2C9 Inhibitor	12092	Metabolism	Classification
CYP2D6 Substrate	664	Metabolism	Classification
CYP2D6 Inhibitor	13130	Metabolism	Classification
CYP3A4 Inhibitor	12328	Metabolism	Classification
CYP3A4 Substrate	667	Metabolism	Classification
Bioavailability	640	Absorption	Classification

Feature Importance

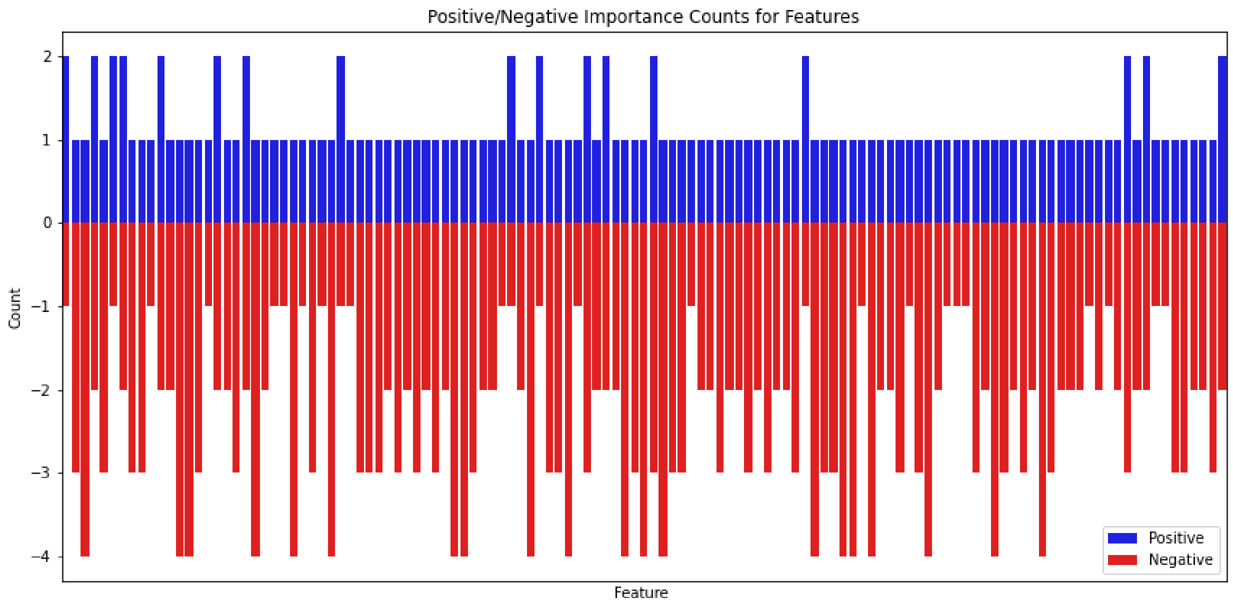

Figure S1: Analysis of feature importance using CYP2D_Substrate. The feature importance experiments are conducted by permutation experiments. Blue bars depict features that decrease model performance while red bars illustrate features increase performance, judged by ROC-AUC changes when removed. This result supports that employing a 5 independent training combined encoding helping the model better recognize important features

Finetuning Hyper-Parameters

Table S3: The parameter specification of the ensemble models.

Datasets	Model Hyper-parameters
BBBP	batch size: 32; learning rate: 1.2e-06;<br>tree depth: 6; num trees: 4; dropout: 0.22013;<br>weight decay: 2.5e-02; gmfu numbers: 3
BACE	batch size: 16; learning rate: 2.9e-07;<br>tree depth: 4; num trees: 5; dropout: 0.33982;<br>weight decay: 2.1e-05; gmfu numbers: 3
HIV	batch size: 32; learning rate: 2.0e-05;<br>tree depth: 7; num trees: 9; dropout: 0.44865;<br>weight decay: 6.5e-07; gmfu number: 5
ClinTox (y1,y2)	batch size: {6,32}; learning rate: {1.7e-07,1.0e-07};<br>tree depth: {3,6} ; num trees: {3,6}; dropout: {0.36797,0.49972};<br>weight decay: {7.5e-07,1.7e-04}; gmfu numbers: {2,2}
AMES	batch size: 32; learning rate: 3.4e-05;<br>tree depth: 8; num trees: 3; dropout: 0.20145;<br>weight decay: 8.6e-03; gmfu numbers: 2
CYP2C9 (Substrate)	batch size: 8; learning rate: 2.9e-6;<br>tree depth: 6; num trees: 6; dropout: 0.46541;<br>weight decay: 8.6e-4; gmfu numbers: 5
CYP2C9 (Inhibitor)	batch size: 20; learning rate: 5.6e-04;<br>tree depth: 4; num trees: 9; dropout: 0.11558;<br>weight decay: 6.3e-02; gmfu numbers: 3
CYP2D6 (Substrate)	batch size: 20; learning rate: 1.4e-07;<br>tree depth: 6; num trees: 9; dropout: 0.44784;<br>weight decay: 6.3e-04; gmfu numbers: 3
CYP2D6 (Inhibitor)	batch size: 20; learning rate: 2.2e-02;<br>tree depth: 4; num trees: 8; dropout: 0.10625;<br>weight decay: 3.4e-05; gmfu numbers: 2
CYP3A4 (Substrate)	batch size: 32; learning rate: 1.8e-04;<br>tree depth: 9; num trees: 8; dropout: 0.14573;<br>weight decay: 1.0e-03; gmfu numbers: 4
CYP3A4 (Inhibitor)	batch size: 40; learning rate: 4.8e-03;<br>tree depth: 9; num trees: 7; dropout: 0.26272;<br>weight decay: 5.2e-04; gmfu numbers: 2
Bioavailability	batch size: 12; learning rate: 1.9e-6;<br>tree depth: 8; num trees: 7; dropout: 0.45363;<br>weight decay: 4.3e-05; gmfu numbers: 3

Model size and parameters count

Table S4: Number of Model Parameters of ChemXTree when training on CYP Datasets

Datasets	BBBP	BACE	HIV	ClinTox	AMES	Bioavailability	**Average**
backbone	120M	667M	230M	2.30B	1.2B	894M	901M
embedding layer	3.0K	11.0K	5.0K	23K	17.0K	17.0K	12.7K
heads	1.0K	39	267	40	518	1.0K	477
Total	120M	667M	230M	2.30B	1.2B	894M	901M

Datasets	CYP2C9 Substrate	CYP2C9 Inhibitor	CYP2D6 Substrate	CYP2D6 Inhibitor	CYP3A4 Substrate	CYP3A4 Inhibitor	**Average**
backbone	2.30B	822M	1.0B	491M	208M	856M	946M
embedding layer	24.0K	14.0K	16.0K	11.0K	7.0K	10.0K	13.7K
heads	72	518	92	74	140	74	162
Total	2.30B	822M	1.0B	491M	208M	856M	946M

Model Optimization

In the process of optimizing hyper-parameters for binary classification models, Bayesian optimization enhances model performance by iteratively adjusting hyper-parameters based on a predefined objective function. This function critically evaluates the balance between training effectiveness and model generalization, which is crucial for ensuring the robustness and applicability of the model to unseen data.

In this approach, the objective function for Bayesian optimization incorporates adjustments to various hyper-parameters such as batch size, weight decay, learning rate, tree depth, number of trees, and dropout rate. These adjustments aim to minimize a composite loss function reflecting both training and validation losses according to a specified weighting scheme.

Specifically, in optimizing a binary classification model, a novel objective function has been devised to calculate the loss as a weighted average of validation loss and training loss, with weights of 0.6 and 0.4 respectively. This weighting underscores the balance between the model's generalization ability (reflected by validation loss) and its learning sufficiency from the training data (reflected by training loss). The rationale for this weighting strategy is to optimize model performance by carefully balancing the trade-off between achieving high predictive accuracy on the training dataset and ensuring the model's generalization to new datasets. Additionally, a penalty factor of 0.1 is introduced to focus to some extent on poorly classified data points in cases of inadequate training. Despite employing 100 optimization iterations, reducing the number of optimizations still achieves state-of-the-art performance of over 90% on benchmark datasets.

References

(1) Xiong, Z.; Wang, D.; Liu, X.; Zhong, F.; Wan, X.; Li, X.; Li, Z.; Luo, X.; Chen, K.; Jiang, H.; others Pushing the boundaries of molecular representation for drug discovery with the graph attention mechanism. *J. Med. Chem.* **2019**, *63*, 8749–8760.

(2) Veličković, P.; Cucurull, G.; Casanova, A.; Romero, A.; Lio, P.; Bengio, Y. Graph attention networks. *arXiv preprint arXiv:1710.10903* **2017**,

(3) Maziarka, Ł.; Danel, T.; Mucha, S.; Rataj, K.; Tabor, J.; Jastrzebski, S. Molecule attention transformer. *arXiv preprint arXiv:2002.08264* **2020**,

(4) Sun, F.-Y.; Hoffmann, J.; Verma, V.; Tang, J. Infograph: Unsupervised and semi-supervised graph-level representation learning via mutual information maximization. *arXiv preprint arXiv:1908.01000* **2019**,

(5) Ying, C.; Cai, T.; Luo, S.; Zheng, S.; Ke, G.; He, D.; Shen, Y.; Liu, T.-Y. Do transformers really perform badly for graph representation? *Advances in Neural Information Processing Systems* **2021**, *34*, 28877–28888.
